# Improving management of hypoglycaemia in children

**DOI:** 10.2471/BLT.21.285586

**Published:** 2021-11-04

**Authors:** Helena Hildenwall, Fatsani Ngwalangwa

**Affiliations:** a Astrid Lindgren Children's Hospital, Paediatric Emergency Care Unit, K 41-43, Huddinge, Karolinska University Hospital, 14186 Stockholm, Sweden.; b Department of Epidemiology and Biostatistics, Kamuzu University of Health Sciences, Blantyre, Malawi.

Studies from countries across all income levels show that the presence of an abnormal blood sugar concentration in sick children is associated with an increased mortality risk.^1^^–^^3^ One of these studies reports that of the 105 hypoglycaemic children admitted to a hospital in United Republic of Tanzania, 42% (44) of the children died.^3^ The World Health Organization (WHO) defines hypoglycaemia as a blood glucose concentration of less than 2.5 mmol/L in non-malnourished children.^4^ Excess mortality has also been observed among children admitted with low blood glucose concentrations, that is a blood glucose concentration above the WHO cut-off of 2.5 mmol/L and with a variable upper limit (depending on the study) of up to 5.0 mmol/L, compared to children with higher blood glucose concentrations.^2^^,^^5^ Accurate management of this patient group is needed to avert the high mortality as well as the potential long-term consequences of low blood glucose concentrations such as neurological deficits, recurrent seizures or cognitive disability.

Several issues surround the management of non-diabetic paediatric hypoglycaemia, especially in low-resource settings. Many health facilities lack the necessary equipment to assess blood glucose concentrations or have an unpredictable inflow of different and often incompatible brands of glucometers and test strips. When available, the commonly used point-of-care glucometers are not precise and tend to be less accurate in the hypoglycaemic range.^6^ Diagnostic accuracy may be further compromised by an interchangeable use in the reporting of glucose concentrations in blood and plasma, with the plasma concentration being 1.11 times higher than the blood concentration. To harmonize results, the International Federation of Clinical Chemistry recommends to always report glucose concentrations in plasma.^7^ While point-of-care devices may lead to both under- and over-diagnosis of hypoglycaemia, waiting for a more exact laboratory measurement of the glucose concentration is rarely an option, considering the urgency of the condition. A higher recommended cut-off concentration to consider treatment would reduce the risk of under-diagnosing hypoglycaemia due to device inaccuracy.

Besides the difficulties in assessing the blood glucose concentration, the diagnosis of hypoglycaemia is not straightforward because the threshold for when low blood glucose concentrations become dangerous is difficult to define and likely varies depending on the overall condition of the child. The optimal treatment of hypoglycaemia also remains unknown. WHO recommends increasing the blood glucose concentration in a hypoglycaemic child without severe malnutrition through the intravenous administration of 5 mL/kg of 10% dextrose. No clinical trials on the mortality impact of the suggested treatment on hypoglycaemic children have been performed, probably because such trials are considered ethically challenging, as they would involve not administering glucose to children with obvious sugar deficit. However, we presume – although it is not reported – that the WHO hypoglycaemia treatment guidelines have been at least partly adhered to in observational studies reporting on the association between admission blood glucose and mortality. If observed hypoglycaemic children indeed received glucose according to the guidelines, this would imply that they still suffered a high case fatality.

The SugarFACT trial assessed whether the WHO hypoglycaemia treatment of children with low blood glucose concentrations (2.5–5.0 mmol/L) reduced the paediatric in-hospital mortality in Malawi.^8^ The recently published null-results from the trial established a 15.5% (50/322) in-hospital mortality in severely sick children aged 1 month to 5 years with low blood glucose concentrations. The combined case fatality among severely ill children in the same hospital who presented with hypo- or low glycaemia (< 5.0 mmol/L) was 21.2% (29/137) compared to 6.9% (70/1011) among children with normoglycaemia of 5.0–10.0 mmol/L and 9.0% (19/211) among children with an admission blood sugar level of 10.0 mmol/L or above.^2^ While the identification and treatment of the underlying cause of the illness will always be a cornerstone for a successful outcome, the results from the SugarFACT trial raises some concerns related to the current treatment recommendations for hypoglycaemia.

First, studies from intensive care settings in high-income countries have elaborated on the importance of glycaemic control in the management of critically ill children since glycaemic variability (that is, swings in blood glucose levels) is associated with increased mortality.^1^ Even isolated episodes of hyperglycaemia and hypoglycaemia are known to be harmful, and hypoglycaemia may go clinically undetected.^9^ Due to the risk of rebound hypoglycaemia and concerns for glycaemic variability, some guidelines are now recommending a smaller dextrose bolus of 2 mL/kg rather than the WHO-recommended 5 mL/kg. Indeed, the use of boluses (a large dose of a substance given by injection for rapid concentration in the bloodstream) in severely ill children has been questioned following results from the Fluid Expansion as Supportive Therapy trial,^10^ where the standard treatment of intravenous fluid boluses for shock in children was found to increase mortality. Boluses may cause too rapid changes in fragile patients whose physiological response fails to adapt. Some small studies from high-income countries suggest that enteral dextrose administration may be the best option for glucose administration in sepsis, inducing the release of intestinal incretin hormones which in turn improves glycaemic control while also decreasing cytokine levels and thereby inflammation.^11^

In resource-poor countries, where health facilities may be unable to ensure intravenous access in severely sick children, sublingual sugar has been promoted as an alternative treatment option for hypoglycaemia. Sublingual dextrose gel in the treatment of neonatal hypoglycaemia has been shown to be beneficial and resulted in fewer episodes of rebound hypoglycaemia compared to intravenous dextrose.^12^ However, the dextrose used in neonates was a specially prepared dextrose gel that is too expensive for low-resource health systems. Therefore, in these settings, the currently recommended treatment of potential hypoglycaemia in the absence of intravenous access is sublingual sugar, that is, sugar mixed with water that is administered under the tongue of the child. Sublingual sugar has been shown to rapidly increase the blood sugar levels and may provide a more physiological increase of blood glucose concentrations, although current evidence is insufficient to translate into clear guidelines.^13^ Frequent repeat measurements combined with sublingual provision of glucose may assist to evaluate and adjust the treatment and could be implemented without the addition of any human or medical resources.^14^ Continuous glucose monitors, initially developed to facilitate the management of diabetes patients, offer another recent opportunity for improved monitoring of admitted children at risk of hypoglycaemia and glycaemic variability. The use of these monitors for admitted children has not yet been well studied, and well-designed cost–effectiveness studies of their potential use to alter the prognosis of hypoglycaemia are needed.

The cut-off WHO uses to define at what level the blood glucose concentration requires to be treated is not based on firm evidence, nor are the timing and need for repeated testing and the optimal treatment. We suggest revising the current guidelines in the WHO *Pocket book of hospital care for children: guidelines for the management of common childhood illnesses, 2nd edition.*

First, given the increased mortality among children with blood glucose concentrations above the WHO hypoglycaemia definition, a higher cut-off than 2.5 mmol/L for defining hypoglycaemia and recommend treatment should be considered. The common lack of glucometers is acknowledged in the guidelines – hence the recommendation of presumptive hypoglycaemia treatment in case of inability to test the blood glucose concentration in a severely sick child. Removal of a hypoglycaemia definition may present a pragmatic and easily applicable approach for resource-constrained health facilities. However, removing the definition also requires better knowledge of the potential harm caused by hypoglycaemia treatment – particularly concerning hyperglycaemia and related changes in electrolyte balance – in certain patient groups. The reasons for a falling blood glucose are diverse but insufficiently documented in low-resource settings. Some conditions such as malaria could benefit from simple replacement of glucose, but other conditions require extended clinical workup for appropriate management.

Second, more research is needed to provide evidence for the best treatment option for low blood glucose concentrations, including the choice of intravenous or sublingual route as well as the optimal dose. Although some studies show promising results from the use of sublingual sugar, well-designed and larger studies with clearly identified patient groups are needed to confirm the presumed benefits. 

Third, and possibly most important, the management guidelines need to cover more than the very initial phase of treating a low blood glucose concentration – starting with recommendations of repeat blood glucose assessments during the first day or days of admission. The technology provided by continuous glucose monitors offers an interesting opportunity for improved management, but repeated point-of-care tests are likely more implementable and acceptable by both guardians and health-care workers. For future evidence-based recommendations, research is needed to better explain the reasons for the high mortality in children with low blood glucose concentrations in resource-poor settings in general, but above all to provide robust evidence on best possible management to avert hypoglycaemia-related morbidity and mortality.

## References

[1] Rake AJ, Srinivasan V, Nadkarni V, Kaptan R, Newth CJ. Glucose variability and survival in critically ill children: allostasis or harm? Pediatr Crit Care Med. 2010 Nov;11(6):707–12. 10.4269/ajtmh.19-0127 20625345

[2] Ngwalangwa F, Phiri CHA, Dube Q, Langton J, Hildenwall H, Baker T. Risk factors for mortality in severely ill children admitted to a tertiary referral hospital in Malawi. Am J Trop Med Hyg. 2019 Sep;101(3):670–5. 10.4269/ajtmh.19-0127 31287044 PMC6726928

[3] Nadjm B, Mtove G, Amos B, Hildenwall H, Najjuka A, Mtei F, et al. Blood glucose as a predictor of mortality in children admitted to the hospital with febrile illness in Tanzania. Am J Trop Med Hyg. 2013 Aug;89(2):232–7. 10.4269/ajtmh.13-0016 23817332 PMC3741242

[4] Pocket book of hospital care for children: guidelines for the management of common childhood illnesses. 2nd edition. Geneva: World Health Organization; 2013. Available from: https://apps.who.int/iris/handle/10665/81170 [cited 2021 Oct 18]. 24006557

[5] Achoki R, Opiyo N, English M. Mini-review: management of hypoglycaemia in children aged 0-59 months. J Trop Pediatr. 2010 Aug;56(4):227–34. 10.1093/tropej/fmp109 19933785 PMC2948531

[6] Hoedemaekers CW, Klein Gunnewiek JM, Prinsen MA, Willems JL, Van der Hoeven JG. Accuracy of bedside glucose measurement from three glucometers in critically ill patients. Crit Care Med. 2008 Nov;36(11):3062–6. 10.1097/CCM.0b013e318186ffe6 18824915

[7] D’Orazio P, Burnett RW, Fogh-Andersen N, Jacobs E, Kuwa K, Külpmann WR, et al.; IFCC-SD-WG-SEPOCT. Approved IFCC recommendation on reporting results for blood glucose: International Federation of Clinical Chemistry and Laboratory Medicine Scientific Division, Working Group on Selective Electrodes and Point-of-Care Testing (IFCC-SD-WG-SEPOCT). Clin Chem Lab Med. 2006;44(12):1486–90. 10.1515/CCLM.2006.275 17163827

[8] Baker T, Ngwalangwa F, Masanjala H, Dube Q, Langton J, Marrone G, et al. Effect on mortality of increasing the cutoff blood glucose concentration for initiating hypoglycaemia treatment in severely sick children aged 1 month to 5 years in Malawi (SugarFACT): a pragmatic, randomised controlled trial. Lancet Glob Health. 2020 Dec;8(12):e1546–54. 10.1016/S2214-109X(20)30388-0 33038950

[9] Madrid L, Sitoe A, Varo R, Nhampossa T, Lanaspa M, Nhama A, et al. Continuous determination of blood glucose in children admitted with malaria in a rural hospital in Mozambique. Malar J. 2017 May 2;16(1):184. 10.1186/s12936-017-1840-x 28464825 PMC5414384

[10] Maitland K, Kiguli S, Opoka RO, Engoru C, Olupot-Olupot P, Akech SO, et al.; FEAST Trial Group. Mortality after fluid bolus in African children with severe infection. N Engl J Med. 2011 Jun 30;364(26):2483–95. 10.1056/NEJMoa1101549 21615299

[11] Shah FA, Kitsios GD, Zhang Y, Morris A, Yende S, Huang DT, et al. Rationale for and design of the study of early enteral dextrose in sepsis: a pilot placebo-controlled randomized clinical trial. JPEN J Parenter Enteral Nutr. 2019 31148210 10.1002/jpen.1608 PMC6884652

[12] Harris DL, Weston PJ, Signal M, Chase JG, Harding JE. Dextrose gel for neonatal hypoglycaemia (the Sugar Babies Study): a randomised, double-blind, placebo-controlled trial. Lancet. 2013 Dec 21;382(9910):2077–83. 10.1016/S0140-6736(13)61645-1 24075361

[13] Ganeshalingam R, O’Connor M. Evidence behind the WHO guidelines: hospital care for children: what is the efficacy of sublingual, oral and intravenous glucose in the treatment of hypoglycaemia? J Trop Pediatr. 2009 Oct;55(5):287–9. 10.1093/tropej/fmp099 19822563

[14] Oxner A, Vellanki M, Myers A, Bangura F, Bangura S, Koroma AM, et al. Reducing mortality from severe malaria in Sierra Leonean children by applying the World Health Organization’s standard malarial protocol with additional sublingual glucose: a continuous quality improvement report. Int J Infect Dis. 2020 Jul;96:61–7. 10.1016/j.ijid.2020.04.046 32339722

